# Utilization of ELISA Using Thioredoxin Peroxidase-1 and Tandem Repeat Proteins for Diagnosis of *Schistosoma japonicum* Infection among Water Buffaloes

**DOI:** 10.1371/journal.pntd.0001800

**Published:** 2012-08-28

**Authors:** Jose Ma. M. Angeles, Yasuyuki Goto, Masashi Kirinoki, Masahito Asada, Lydia R. Leonardo, Pilarita T. Rivera, Elena A. Villacorte, Noboru Inoue, Yuichi Chigusa, Shin-ichiro Kawazu

**Affiliations:** 1 National Research Center for Protozoan Diseases, Obihiro University of Agriculture and Veterinary Medicine, Obihiro, Hokkaido, Japan; 2 Laboratory of Molecular Immunology, Department of Animal Resource Sciences, Graduate School of Agricultural and Life Sciences, The University of Tokyo, Tokyo, Japan; 3 Laboratory of Tropical Medicine and Parasitology, Dokkyo Medical University, Tochigi, Japan; 4 Department of Parasitology, College of Public Health, University of the Philippines, Manila, Philippines; University of Edinburgh, United Kingdom

## Abstract

**Background:**

The presence of animal reservoirs in *Schistosoma japonicum* infection has been a major obstacle in the control of schistosomiasis. Previous studies have proven that the inclusion of control measures on animal reservoir hosts for schistosomiasis contributed to the decrease of human cases. Animal surveillance should therefore be included to strengthen and improve the capabilities of current serological tests.

**Methodology/Principal Findings:**

Thioredoxin peroxidase-1 (SjTPx-1) and four tandem repeat proteins (Sj1TR, Sj2TR, Sj4TR, Sj7TR) were initially evaluated against human sera. The previous test showed high sensitivity and specificity for antibody detection against SjTPx-1 and Sj7TR. In this study, the immunodiagnostic potential of these recombinant proteins was evaluated using enzyme-linked immunoassay on 50 water buffalo serum samples collected in Cagayan, the Philippines as compared with the soluble egg antigen (SEA). For specificity, 3 goat serum samples positive with *Fasciola hepatica* were used and among the antigens used, only SEA showed cross-reaction. Stool PCR targeting the *S. japonicum* 82 bp mitochondrial NAD 1 gene was done to confirm the true positives and served as the standard test. Twenty three samples were positive for stool PCR. SjTPx-1 and Sj1TR gave the highest sensitivity among the recombinant proteins tested for water buffalo samples with 82.61% and 78.26% respectively which were higher than that of SEA (69.57%).

**Conclusions/Significance:**

These results prove that SjTPx-1 works both for humans and water buffaloes making it a good candidate antigen for zoonotic diagnosis. Sj1TR showed good results for water buffaloes and therefore can also be used as a possible candidate for detecting animal schistosome infection.

## Author Summary

Schistosomiasis remains to be a public health problem in 76 endemic countries in spite of control efforts that have been done. Among the major causative agents of schistosomiasis, only *Schistosoma japonicum* is known to be zoonotic. However, the role of animal reservoir hosts has not been given much importance which might be the main hindrance in the possible elimination of the disease. In addition, animal surveillance is not part of the current schistosomiasis control program in most of the endemic countries. This study reports the use of recombinant proteins in ELISA for detecting the infection in water buffaloes. These antigens were previously used against humans and showed that SjTPx-1 and Sj7TR can be a good diagnostic antigen. Using the stool PCR as the standard test, SjTPx-1 and Sj1TR were shown to work on the water buffaloes better than the conventional SEA. These antigens can both be useful in the development of intensified animal surveillance for schistosomiasis.

## Introduction

Intensified disease surveillance has become an essential public health instrument in providing necessary information for monitoring the disease and evaluating control measures. Schistosomiasis is considered as a neglected disease caused by *Schistosoma japonicum* in China and Southeast Asia, *S. haematobium* in the Middle East and Africa and *S. mansoni* in Africa. Among them, only *S. japonicum* is known to infect both humans and more than 40 other mammals [1] which complicate the control of the disease. Inclusion of zoonotic surveillance in national control programs in endemic countries might be a necessary tool for the control and elimination of schistosomiasis japonica. Researches have shown how intervention involving animal reservoirs can reduce *S. japonicum* infection in humans [2], [3]. Simultaneous treatment of water buffaloes and human has proven to be effective as seen in a five-year praziquantel-based intervention study done around the Poyang Lake in Jiangxi Province, China [2]. However, animal surveillance for schistosomiasis has not yet been fully developed.

In China, a nationwide schistosomiasis survey in 1995 established the high prevalence of *S. japonicum* in water buffalo (9.6%) and cattle (7.2%) [4], showing how important these animals are as reservoir hosts. In Indonesia, domestic animals such as water buffaloes and wild animals were found to be infected with schistosomes (10%) [5]. In the Philippines, a variety of animal reservoir hosts such as rats, cats, dogs, pigs, cattle and water buffaloes were found to be potential hosts for schistosomiasis using different parasitological and immunological assays [6]–[8]. Among these hosts, water buffaloes had the lowest prevalence of infection [8] and showed no significant role in the *S. japonicum* transmission to humans according to the mathematical modeling done on these prevalence data [9]. A recent study however in one endemic area in Leyte showed prevalence in water buffaloes as high as 51.5% using the highly validated real-time polymerase chain reaction [10]. This may prove that water buffaloes have a major contribution to the transmission of schistosomiasis in the Philippines.

Animal schistosome infection has been usually diagnosed through direct parasitological techniques including Kato-Katz technique and miracidial hatching. The quantitative Kato-Katz fecal smear is simple, practical and useful in quantifying eggs [11], [12] and is considered by the World Health Organization as the gold standard method for diagnosing schistosomiasis [13]. However, this method is labor-intensive, requires skilled personnel, has low sensitivity in low prevalence endemic areas [14], [15] and seven repeated Kato-Katz examinations coupled with miracidial hatching are needed to reach its maximal sensitivity [16]. On the other hand, molecular detections such as polymerase chain reaction (PCR) are highly sensitive and specific, but they are costly and require expensive equipment. Furthermore, current serological tests utilizing crude antigens like soluble egg antigen-enzyme-linked immunosorbent assay (SEA-ELISA) and circum-oval precipitin test (COPT) cause cross-reactions leading to misdiagnosis. Hence there is a need for the development of an easier, more sensitive and specific test for schistosomiasis.

In a previous study, thioredoxin peroxidase-1 (SjTPx-1, GeneDB accession no. Sjp_0095720.1) and four tandem repeat proteins (TRP) namely Sj1TR, Sj2TR, Sj4TR and Sj7TR (GeneDB accession nos. Sjp_0099630, Sjp_0086200, Sjp_0059850, Sjp_0110390 respectively) were evaluated against human sera [17]. SjTPx-1 and Sj7TR both showed high sensitivity and specificity making them promising diagnostic antigens for human schistosomiasis. Using ELISA, these recombinant proteins were tested on water buffaloes and the results were compared with stool PCR assay and the conventional SEA-ELISA and COPT. This study therefore examined the immunodiagnostic potential of the recombinant antigens in water buffaloes which might lead to the development of a more reliable and accurate diagnostic test for animal schistosomiasis. Strengthening the diagnostic test is crucial in both the human and animal schistosome infection surveillance in areas where elimination is in sight and might be vital in the prevention of emergence and re-emergence of schistosomiasis japonica leading to the possible control of this neglected parasitic disease.

## Materials and Methods

### Samples

Serum and stool samples were taken from 50 water buffaloes in Gonzaga, Cagayan, the Philippines. Stool samples collected by intrarectal means from water buffaloes were placed in code-labeled cups and stored with 10% neutralized formalin until processing. None of the stools were found positive for *S. japonicum* eggs using the formalin-ether concentration technique (FECT). Non-endemic negative control sera were taken from 18 water buffaloes in Nueva Ecija and Batangas in the Philippines. All the owners of the water buffaloes were informed about the study and gave consent to use their water buffaloes in this study. Sera positive for *Fasciola hepatica* were collected from experimentally infected goats (*N* = 3). They were diagnosed through the detection of the parasite in the stool. This study was done according to ethical guidelines for the use of animal samples permitted by Animal Care and Use Committee, Dokkyo Medical University (Permit No. 0029) in accordance with the Guidelines for the Care and Use of Laboratory Animals, Dokkyo Medical University, The Law Concerning Kind Treatment and Management of Animals (Law No. 221) and Japanese Government Notification on Feeding and Safe-keeping of Laboratory Animals (No. 6), as well as by Obihiro University of Agriculture and Veterinary Medicine (Permit No. 23–153).

### Stool DNA Extraction

FECT was done prior to DNA extraction to maximize the quantity of schistosome eggs in the collected stool if positive and to remove fecal debris. Although formaldehyde is known to degrade DNA, DNA extraction was not deterred since neutral-buffered formaldehyde was used [18], [19] and the PCR target is less than 400 bp [20]. DNA extraction from stool samples was done using QIAamp DNA Stool Mini Kit (QIAGEN Inc., Valencia, CA) according to the manufacturer's protocol and stored at −20°C until use. DNA was also extracted from cattle stool in non-endemic area (Obihiro, Hokkaido, Japan) to serve as the negative control.

### Stool PCR

PCR was done on the stool samples collected from 50 water buffaloes targeting the 82 bp mitochondrial NADH dehydrogenase I gene (SjND1) [21]. The primer set SjND1 forward 5′-TGR TTT AGA TGA TTT GGG TGT GC3′ and reverse 5′ AAC CCC CAC AGT CAC AGT CAC TAG CAT AA3′ was used according to a previous research [22]. Twenty microliters of reaction mixture contained 2 µl of buffer, 0.6 µl of 1.5 mM MgCl_2_, 1.6 µl of 2.5 mM dNTP, 0.4 µl of each 20 pmol/µl primer, 0.2 µl of 5 U/µl *Taq* DNA polymerase (Takara, Otsu, Japan) and 1 µl of template. The conditions for PCR were as follows: 95°C for 10 mins, followed by 40 cycles of 95°C for 15 secs, 60°C for 1 min, 72°C for 1 min, and a final extension of 72°C for 10 min. The PCR was performed using Veriti 96 Well Thermal Cycler (Applied Biosystems, Carlsbad, CA). The PCR products were separated by electrophoresis in 2.5% agarose gel and visualized by ethidium bromide staining. PCR reactions were done in triplicates for every stool sample and a sample is regarded as positive when at least one reaction was positive.

### Recombinant Antigen Preparation

Recombinant molecules of SjTPx-1 and the four TRPs from *S. japonicum* used in this study were prepared as previously described [17]. In brief, SjTPx-1 was cloned using PCR from *S. japonicum* Yamanashi strain adult worm cDNA while the nucleotides coding a partial tandem repeat domain of the 4 TRPs were synthesized by GenScript USA Inc. (Piscataway, NJ). The genes were then digested with their respective restriction enzymes, inserted into the pET28 vector (EMD Biosciences, San Diego, CA) and transfected into *Escherichia coli* BL21 grown in SOB medium (Sigma-Aldrich, St. Louis, MO). The recombinant proteins were recovered using the Ni-NTA agarose (Qiagen Inc., Valencia, CA), dialyzed and eluted with 20 mM Tris, pH 8.0. The integrity and purity of the proteins were evaluated by 15% polyacrylamide gel electrophoresis (SDS-PAGE) under denaturing conditions and subsequent Coomassie Brilliant Blue staining. The concentration of each expressed protein was measured using the BCA Protein Assay (Thermo Scientific, Rockford, IL).

### Serological Tests

#### (i) COPT

COPT was performed as previously described [23]. One drop of serum sample was mixed on a slide with approximately 5 µg of lyophilized schistosome eggs. The eggs were collected from a rabbit infected with *S. japonicum* for 12 weeks. A cover slip was then placed over a nail polish ring surrounding the egg suspension. The slides were then sealed with paraffin and incubated at 37°C for 48–72 h. Bleb and segment formation for positive samples were detected under the microscope.

#### (ii) ELISA

The conventional ELISA was done according to a previously described method [24] with slight modifications. Horseradish peroxidase (HRP)-conjugated Protein G (Rockland Inc., Gilbertsville, PA) served as the secondary antibody in this study and 3,3′,5,5′-tetramethylbenzidine (KPL, Gaithersburg, MD) was used as the substrate for HRP. Ninety-six wells microplates (Nunc Maxisorp, Thermo Fisher, Rockland, IL) were sensitized separately with SEA (1 µg/well) or each of the recombinant proteins (200 ng/well). Proteins were diluted with carbonate/bicarbonate buffer at pH 9.6. After blocking with 1% bovine serum albumin (BSA) in phosphate buffered saline with 0.05% Tween 20 (T-PBS) (T-PBS-1%BSA), the serum samples were placed on the antigen-coated wells. The test sera (0.1 ml) were diluted 200-fold in T-PBS-1%BSA while the secondary antibody (0.1 ml) was diluted in 10,000-fold. Optical density (OD) at 450 nm was determined using a microplate reader (MTP-500, Corona Electric, Tokyo, Japan). Each ELISA reaction was performed with positive (8-weeks post-infected rabbit serum) and negative controls (diluting buffer). All the tests were done in triplicates and data represent mean values. The cut-off value was calculated as the mean absorbance value of the 18 negative controls plus 3 standard deviations. A sample was considered positive when the mean absorbance value of each sample was higher than the cut-off value.

### Statistical Analysis

The validity of the ELISA assays using the recombinant proteins was estimated by the sensitivity, specificity and predictive values using the stool PCR as the reference standard. Kappa value was used to estimate the agreement between the antigens [25]. To test for the statistical significance of the difference between the mean OD values of the PCR positive and PCR negative samples on the ELISA using the crude and recombinant antigens, two-tailed p-value was calculated using unpaired *t* test with 95% confidence interval.

## Results

### Stool PCR

**Figure 1:**
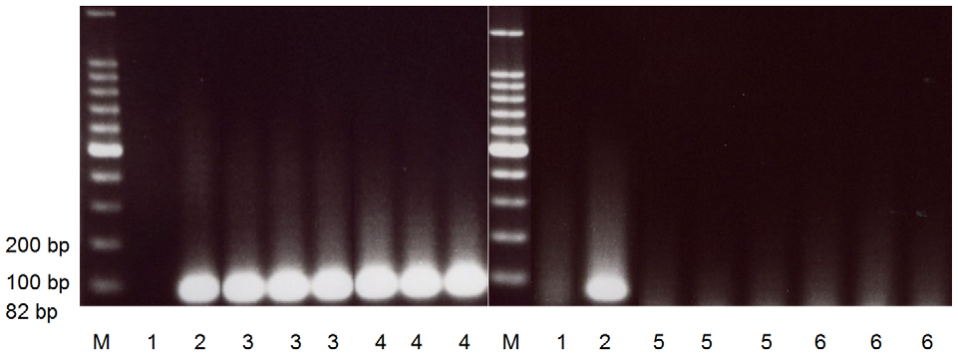
Gel electrophoresis of the stool PCR for water buffaloes targeting the *Schistosoma japonicum* NAD 1 gene. *M*, marker. *Lane 1*, negative control (stool DNA from non-endemic cattle). *Lane 2*, positive control (*S. japonicum* adult DNA template). *Lane 3–6*, water buffalo stool samples done in triplicates. Positive control, lanes 3 and 4 show positive results with bands at 82 bp while none is seen on negative control, lanes 5 and 6.

Stool PCR was performed to serve as the standard test by determining the positives for *S. japonicum* infection. Stool DNA from a non-endemic cattle served as the negative control and *S. japonicum* DNA template served as the positive control. Of the 50 water buffalo samples, 23 were positive. As seen on Figure 1, a band having approximately 82 bp was found in the positive samples while none on the negative samples. The band was also seen in the positive control but not in the negative control.

### Serological Tests

COPT was done initially on the 50 water buffalo samples for the purpose of comparing it with the ELISA using the recombinant proteins. Seventeen samples turned out to be positive as shown by bleb or segment formation after 48 h incubation. All of the samples positive for COPT were also PCR positive.

**Figure 2:**
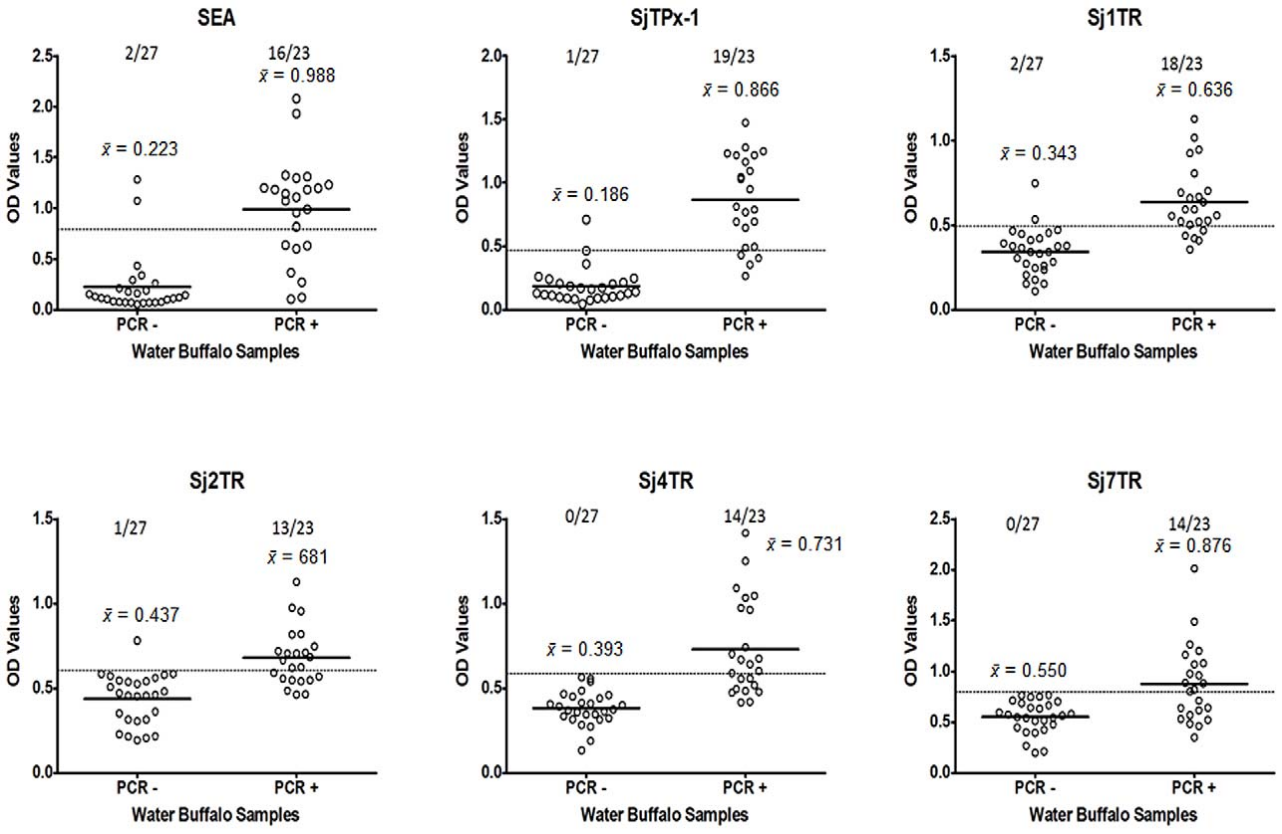
Difference in OD values among stool PCR negative and positive in ELISA using SEA and the recombinant proteins. The graph shows that SjTPx-1 and Sj1TR have the highest number of positives among the PCR positive samples. PCR negative samples that are positive for ELISA using the recombinant proteins show OD values minimally higher than the cut-off values. Mean OD values for each set were also shown.

The ELISA was performed using sera from 50 water buffaloes from an endemic area in the Philippines. Cut-off values were calculated using 18 water buffalo serum samples from non-endemic areas in the Philippines. Twenty samples were positive for both SjTPx-1 and Sj1TR, 18 for SEA and 14 samples for Sj2TR, Sj4TR and Sj7TR. As shown on Table 1, 16 out of the 18 SEA positive, 19 out of the 20 SjTPx-1 positive and 18 out of the 20 Sj1TR positive samples were also PCR positive. There were 2 PCR negative samples detected only by SEA and Sj1TR, and of which, 1 was also detected by SjTPx-1. Furthermore, there were 4 samples detected only by PCR and negative for all the recombinant proteins and SEA. The mean OD values for PCR negative were lower than that of the PCR positive samples (Figure 2) for the crude and the recombinant antigens. The p-values obtained to show the significance of the difference between the mean OD values of PCR positive and PCR negative samples were all less than 0.05 and were considered statistically significant (data not shown).

**Table 1:** Results tally of the samples for *Schistosoma japonicum* using serological tests and stool PCR.

	COPT		SEA		SjTPx-1		Sj1TR		Sj2TR		Sj4TR		Sj7TR	
	Pos	Neg	Pos	Neg	Pos	Neg	Pos	Neg	Pos	Neg	Pos	Neg	Pos	Neg
**Negative by PCR**	0	27	2	25	1	26	2	25	1	26	0	27	0	27
**Positive by PCR**	17	6	16	7	19	4	18	5	13	10	14	9	14	9

To test for specificity, 3 serum samples from goats experimentally infected with *Fasciola hepatica* were also used. Only SEA showed cross-reaction with 2 samples having high OD values (data not shown).

Based on the statistical analysis, SjTPx-1 and Sj1TR showed high agreement with the stool PCR done on the samples based on the kappa values as seen in Table 2. The specificity and the positive predictive values of these 2 recombinant proteins were higher than those of SEA.

**Table 2:** Statistical analysis of the ELISA results of SEA and the recombinant proteins.

Antigen^a^^,^ b	Sensitivity (%)	Specificity (%)	PPV (%)	NPV (%)	Kappa^c^
SEA	69.57	86.67	80	78.79	0.571
SjTPx-1	82.61	96.67	95	87.88	0.805
Sj1TR	78.26	93.33	94.74	84.85	0.765
Sj2TR	56.52	96.67	92.85	74.36	0.557
Sj4TR	60.87	100	100	76.92	0.638
Sj7TR	60.87	100	100	76.92	0.638

a Stool PCR served as the reference standard.

b ELISA = enzyme-linked immunosorbent assay; SEA = soluble egg antigen; PPV = positive predictive values; NPV = negative predictive values.

c Kappa value of SjTPx-1 showed high agreement with the stool PCR based on the previously reported criteria set [25].

## Discussion

The lack of importance given to the role of animal hosts in the transmission of *S. japonicum* has turned into a loophole in the control efforts for schistosomiasis. Efficient and highly sensitive diagnostic tools for animal surveillance should be employed as a strong support in ensuring control of the parasitic infection among the reservoir hosts. This study aims to determine the possible use of the recombinant antigens in the diagnosis of schistosomiasis among the water buffaloes. Results of the study are expected to contribute to clearer insights in the role of this animal in the transmission of the disease.

SjTPx-1, which has a sensitivity of 85.71% for humans [17] and 84.0% for cattle [26] in previous studies, showed a comparable 82.61% sensitivity among the water buffaloes. However, it should be noted that the infection standard between these studies are different, with stool PCR confirmed samples used in this study and microscopy confirmed samples in the previous studies. But despite this difference, SjTPx-1 showed good immunodiagnostic potential in all these studies and therefore might be an effective diagnostic antigen candidate for both humans and animals. Furthermore, Sj1TR performed better in water buffaloes (78.26%) than in humans (68.57%) while Sj7TR did not show good antigenicity in water buffaloes (60.87%) as it did in humans (85.71%). These differences in antigenicity can be explained by the differences in immune responses among various host species.

On the other hand, results showed that SEA has lower sensitivity than SjTPx-1 and Sj1TR, and causes cross-reaction with *F. hepatica* positive samples. Both the conventional SEA-ELISA and COPT therefore are not adequate enough to properly diagnose cases of schistosomiasis. Given this and the difficulty in scaling up production of SEA for mass screening, the use of recombinant proteins has proven to be a good alternative for schistosomiasis diagnosis.

In this study, we used stool PCR as the standard test instead of stool microscopy. Coprological methods such as Kato-Katz technique have been the commonly accepted gold standard in schistosomiasis diagnosis. However, it will be difficult to detect schistosome eggs in the stool of large animals due to the size of their excreta which might affect the sensitivity of the test. The adequacy of the stool PCR in diagnosing true positives has been already validated by a previous study done among water buffaloes in the Philippines [10]. Their results showed a marked difference in the number of positive water buffaloes with 51.5% prevalence for stool PCR as against to the 3.7% prevalence using the coprological tests including DBL and Kato-Katz technique. Stool PCR has been tested also in other helminthic [27] and protozoan [28] infection and was found to possess higher sensitivity and specificity as compared to stool microscopy.

Furthermore, samples which were PCR negative and positive for the recombinant protein-based ELISA should be investigated further. It was shown in the initial assessment of the stool PCR that its sensitivity can be affected by the degree of infection [22]. For example, in an infection higher than 10 schistosome eggs per gram (epg) of stool, the sensitivity can go as high as 95 to 100%. The sensitivity goes down to 78 to 85% when the infection is less than 10 epg. It is therefore very important to adjust the diagnostic capabilities of the recombinant proteins to detect cases even in very low infections which are undetectable even with molecular techniques such as PCR. Furthermore, the extent of time that the antibodies against these recombinant proteins will be present in the blood circulation should also be analyzed. It was widely known that one of the limitations of antibody-based serological tests is that it cannot distinguish past and present infection. In addition, the infection in water buffaloes is self-limiting [29] which further complicates the possible diagnosis of active infection. In the previous paper using these recombinant proteins [17] however, serum samples from human individuals collected one year after treatment with praziquantel tested negative for the recombinant antigens. This somehow suggests that the recombinant antigens might be used to detect current infection in humans. However, it was not yet studied in animals and it will be very useful if these recombinant antigens can also be used to diagnose present animal infection as well.

As this study proved the serological applicability of SjTPx-1 and Sj1TR in water buffaloes, this might be also used in the development of rapid immunochromatographic tests that can detect animal schistosome infection in the field. Although the possible reservoir animal hosts in endemic areas can also undergo mass drug administration as previously done in China, serological tests utilizing these recombinant proteins will be useful in epidemiological studies and surveillance of animal infection in areas that have reached elimination level. It was reported that inappropriate surveillance system was one of the factors attributed to the re-emergence of schistosomiasis in one province in China [30]. Infection rate among the cattle in that province was reported to have reached as high as 22.3%. Mammalian reservoir hosts might serve as the sentinel population in schistosomiasis transmission as they have the potential to be the key source of *S. japonicum* infection in re-emerging regions [31]. The World Health Organization noted that case detection will be a problem when elimination of the disease is at hand [32]. Environmental monitoring was said to be important in knowing the scale of the transmission mechanism in such low transmission environment [33]. Strengthening therefore the diagnostic capabilities of serological tests might be one of the vital keys in the possible prevention of such re-emergence of the disease.

On the other hand, the emergence of schistosomiasis in new endemic foci is also a threat to the possible elimination of the disease. The site used in this study, Cagayan Valley, was not known to be endemic of schistosomiasis until 2002 [34]. Based on the results of this study, schistosome infection among water buffaloes has a positivity rate ranging from 24% to 46% using ELISA and stool PCR respectively. Animal infection might play a big role in the transmission of the disease in that area. A more specific and sensitive animal surveillance is therefore also needed to prevent spreading of the disease in other areas. Furthermore, the recombinant antigens used in this study should also be tested against other animals like dogs, pigs and rats as previous studies showed that they are also important reservoir hosts for *S. japonicum* [9], [35]. This will also provide a more realistic epidemiological picture of the disease which is very important in the control program.

In the future studies, the use of these recombinant antigens should be also validated in areas with different levels of endemicity both for humans and animals. This stage is relevant as the challenge now is to optimize the diagnostic use of these recombinant antigens to the different stages of active control. Appropriate diagnostic tools were strongly needed to evaluate effectiveness of community interventions, verify local disease elimination and detect resurgence of the disease at the earliest time possible [36].
